# Comprehensive analysis of histone methylation modification regulators for predicting prognosis and drug sensitivity in lung adenocarcinoma

**DOI:** 10.3389/fcell.2022.991980

**Published:** 2022-10-03

**Authors:** Ying Ye, Li Li, Qinjin Dai, Yan Liu, Lin Shen

**Affiliations:** ^1^ Department of Cardiothoracic Surgery, The Second Affiliated Hospital of Chongqing Medical University, Chongqing, China; ^2^ Guangzhou Women and Children’s Medical Center, Guangzhou Medical University, Guangzhou, China

**Keywords:** lung adenocarcinoma, histone methylation, gene signature, prognostic model, drug sensitivity

## Abstract

Histone methylation is an epigenetic modification regulated by histone methyltransferases, histone demethylases, and histone methylation reader proteins that play important roles in the pathogenic mechanism of cancers. However, the prognostic value of histone methylation in lung adenocarcinoma (LUAD) remains unknown. Here, we found that LUAD cases could be divided into 2 subtypes by the 144 histone methylation modification regulators (HMMRs), with a significant difference in OS time. Ninety-five of the HMMRs were identified as differentially expressed genes (DEGs) between normal and tumor samples, and 13 of them were further discovered to be survival-related genes (SRGs). By applying the least absolute shrinkage and selector operator (LASSO) Cox regression, we constructed an 8-gene-based risk signature according to the TCGA (training) cohort, and the risk score calculated by the signature was proven to be an independent factor in both the training and validation cohorts. We then discovered that the immune functions were generally impaired in the high-risk groups defined by the HMMR signature (especially for the DCs and immune check-point pathway). Functional analyses showed that the DEGs between the low- and high-risk groups were related to the cell cycle. The drug sensitivity analysis indicated that our risk model could predict the sensitivity of commonly used drugs. Moreover, according to the DEGs between the low- and high-risk groups, we discovered several new compounds that showed potential therapeutic value for high-risk LUAD patients. In conclusion, our study demonstrated that HMMRs were promising predictors for the prognoses and drug therapeutic effects for LUAD patients.

## Introduction

Lung cancer is the most common malignancy in the world, and its morbidity and mortality have increased rapidly over the years. There are approximately 2 million new cases of lung cancer worldwide and 1.76 million deaths directly or indirectly due to lung cancer per year, of which more than 70% are non-small cell lung cancer (NSCLC) (Thai et al., 2021). Lung adenocarcinoma (LUAD) gradually became the most common histological type, accounting for approximately 50%–70% of NSCLCs (Kinoshita et al., 2016). Despite advances in molecular biology and the development of new drugs in recent years, the overall survival rate of lung cancer remains poor, with a 5-year survival rate of less than 15% (Siegel et al., 2020). The initial symptoms of LUAD are often not obvious, and by the time the disease becomes apparent, it is already in an advanced stage. LUAD is treated with radiotherapy, chemotherapy, immune checkpoint inhibitor therapy, and molecular targeted therapy, of which molecular targeted therapy has achieved excellent results in recent years but is prone to treatment resistance (Drosten and Barbacid, 2022; Seguin et al., 2022). Lung cancer is often accompanied by abnormal expression of multiple genes, and recent studies have indicated that multigene-targeted treatments have favorable therapeutic effects (Piñeiro-Yáñez et al., 2018). Thus, an in-depth understanding of the molecular pathogenesis and explorations of novel biological markers affecting lung cancer prognosis for individualized treatment are of great significance to improve the overall survival of LUAD patients.

Epigenetic modifications are the transcriptional regulation of a gene without altering its DNA sequence, which are widely involved in the occurrence and development of tumors and have played important roles in the diagnosis, prognosis, and therapeutic drug development of tumors in recent years (Mohammad et al., 2019; Zhao et al., 2021). Histones are proteins with highly conserved sequences, including H1, H3, H2A, H2B, and H4, which are bound to DNA to form chromatin in the nucleus, and their N-terminal amino acid residues can be modified by methylation, acetylation, phosphorylation, ADP-ribosylation, and ubiquitination (Millán-Zambrano et al., 2022). Among them, histone methylation is a very important epigenetic modification. It can regulate gene transcription and translation by affecting the structure and relaxation of chromatin, which is involved in various biological processes, such as heterochromatin formation, X chromosome inactivation, gene imprinting, and DNA damage repair (Bhat et al., 2021). Histone methylation occurs at the lysine and arginine residue sites in the N-terminal tail of H3 and H4 and is dynamically catalyzed by histone methyltransferases (HMTs) and histone demethylases (HDMs) (Huo et al., 2021). The histone lysine residues can be mono-, di- or trimethylated, while the arginine residues can be mono-, di- and asymmetrically demethylated (Greer and Shi, 2012). HMTs can be classified into three functional enzyme families: SET domain-containing lysine methyltransferases, the non-SET domain DOT1L lysine methyltransferase PRDM family, and the arginine methyltransferase PRMT family (Di Tullio et al., 2022). Aberrant expression of histone methyltransferases is detected in many human tumors, participating in cell cycle regulation, epithelial-mesenchymal transition (EMT), apoptosis, and other oncogenic mechanisms and has been proven to be closely associated with the prognosis of various malignancies (Singh, 2019). The methylation level of histones is also regulated by HDMs, which can be divided into two major families, namely, lysine-specific demethylases (LSD) and histone demethylases containing the JMJC structural domain (He et al., 2022). The LSD protein family consists of *LSD1* and *LSD2*, which can both catalyze H3 lysine 4 (H3K4me1/2) to form nonmethylated lysine, in addition, *LSD1* can be involved in the demethylation of histones H3 lysine 9 (H3K9me1/2), and these processes are closely related to the development of various diseases (Shi and Tsukada, 2013). Other HDMs containing JMJC structural domains, including KDM2 to 7, are iron- and 2-ketoglutarate-dependent dioxygenases that can remove the methylation of H3K9me1/2, H3K27me1/2 and H4K20me1 associated with transcriptional silencing (Markolovic et al., 2016; Yoo et al., 2020). Generally, HMTs and HDMs dynamically maintain the balance of the histone methylation level, whereas the abnormal methylation level contributes to tumorigenesis. In addition, there is a large class of proteins or structural domains called histone methylation reader proteins (HMRPs) that specifically recognize different types of histone modifications and play important roles in epigenetic regulation mediated by methylation modifications as adaptors (Cheng et al., 2020). Recently, numerous studies have confirmed that HMT/HDM inhibitors can be used as potential antitumor drugs with promising therapeutic effects; however, there is not enough research on the reader domains. Therefore, exploring the functions and prognostic values of histone methylation modification regulators (HMMRs), including the study of “readers”, is necessary for the development of highly selective targeted drugs.

Based on the existing findings, we know that histone methylation plays an important role in tumors; however, its specific functions in LUAD have been less studied. For this reason, we performed a study to comprehensively understand the expression patterns of HMMRs in LUAD and explore the prognostic value of these regulators, as well as to make targeted therapies more feasible.

## Materials and methods

### Data collection

The transcriptome sequencing data, including 54 normal lung tissues and 497 LUAD samples, and the corresponding clinical information were obtained from The Cancer Genome Atlas (TCGA) database (downloaded at https://portal.gdc.cancer.gov/). An external validation cohort containing the transcriptome data and the clinical features of 398 LUAD patients was downloaded from the Gene Expression Omnibus (GEO) database (https://www.ncbi.nlm.nih.gov/geo/, GSE72094). The RNA-seq data were all downloaded as fragments per kilobase million (FPKM), and the “Scale” function was used to normalize the expression data of each gene before external validation. The histone methylation modification regulators (HMMRs) were acquired from the WERAM 1.0 database (Writers, Erasers, and Readers protein of Histone Acetylation and Methylation system database; http://weram.biocuckoo.org/) in December 2021, and after filtering out duplicate genes, 144 of them were retained for further analysis.

### Consensus clustering analysis

To explore whether there were connections between the expression profiles of HMMRs and LUAD subtypes, we employed the “Consensus Cluster Plus” package in Bioconductor to classify the tumors in the TCGA cohort. Kaplan–Meier survival analysis was used to compare the survival status among tumor subtypes.

### Identification of the differentially expressed genes and survival-related genes

We applied the “limma” R package to identify the differentially expressed genes (DEGs) between tumor and nontumor samples following the criteria of false discovery rate (*FDR*) < 0.01. A volcano plot was established by employing the “GEOquery,” “limma,” “ggplot2,” “ggrepel,” and “ggthemes” R packages to show the expression levels of the HMMRs.

Combined with the survival information of patients in the TCGA cohort, we evaluated the prognostic value of each HMMR, and the survival-related genes (SRGs) were detected by the univariate Cox regression model with *p* < 0.05 by using the “survival” R package. A Venn diagram accomplished by applying the “VennDiagram” R package was utilized to screen the intersecting genes for further analysis. Spearman correlation analysis was conducted by employing the “reshape2” R package, while forest plots and violin plots were established by the “forestplot” and “vioplot” R packages, respectively.

### Construction and validation of a risk model

To develop an HMMR-related risk gene signature, the TCGA cohort was treated as the training set, and a GEO cohort (GSE72094) was used as the external validation set. Those intersecting genes were chosen for developing the prognostic risk signature by applying the least absolute shrinkage and selection operator (LASSO) Cox regression model, which was involved in the “glmnet” R package. Following the minimum criteria and simulating more than 1000 times, 8 genes with nonzero coefficients were retained. Based on the coefficient of each gene, the risk score was calculated by the following formula: Risk Score = $\sum_{i}^{8}C_{i}E_{i}$ (C: coefficients, E: expression levels). In terms of the risk score, patients were divided into low- and high-risk subgroups, and to show the distinctions, we applied principal component analysis (PCA) and t-distributed stochastic neighbor embedding (t-SNE), which were performed by the “ggplot2” and “Rtsne” R packages, respectively. To evaluate the sensitivity and specificity of our risk model, we constructed a time-dependent receiver operating characteristic curve (ROC) by applying the “timeROC” R package. The risk scores of patients in the validation cohort were obtained from the same formula, and these patients were also classified into 2 subgroups to make comparisons.

### Prognostic value of the risk score

We next employed univariate and multivariable Cox regression models conducted by the “survival” R package to assess the prognostic value of the risk model. The clinical characteristics, including age, sex, and tumor stage (I to IV), in combination with the risk score, were included in the regression models. These variables were then utilized to construct a nomogram with the “survival” and “rms” R packages. In addition, we set up the calibration curves (applying the “foreign” R package) to evaluate the consistency between ideal and actual predicting outcomes conducted by the regression model.

### Immune status comparisons

Based on the molecular markers, we identified 16 types of immune cells and 13 immune-related pathways, which are provided in Supplementary Table S1. Single-sample gene set enrichment analysis (ssGSEA), which was performed by the “gsva” R package, was used to calculate the enrichment scores of immune cells and to estimate the activity of immune-related pathways between subgroups.

### Functional enrichment analysis of differentially expressed genes

The DEGs between the low- and high-risk subgroups were identified by the criteria of |log_2_ FC|≥1 and *FDR* < 0.05. To better understand the specific functions of the DEGs, we performed Gene Ontology (GO) and Kyoto Encyclopedia of Genes and Genomes (KEGG) analyses (utilizing the “cluster Profiler” R package).

### Drug sensitivity analysis and cMap analysis

The drug sensitivity analysis was accomplished by the “pRRophetic” R package. The pRRophetic algorithm constructs a ridge regression model to predict drug IC50 based on GDSC (Genomics of Drug Sensitivity in Cancer) and gene expression profiles. The whole gene expression profiles were compared to determine the IC50 of common therapeutic drugs between the low- and high-risk subgroups in the TCGA cohort. We used the Connectivity Map (cMap) online tool (https://clue.io/) to screen novel efficient drugs according to the particular gene expression patterns (up- and downregulated genes) in the high-risk group. The connectivity score ranged from -100 to 100 and indicates the degree of correlation between a compound and the gene expression patterns. Positive connectivity scores demonstrate that the compounds promote the gene expression patterns, while negative scores indicate that the compounds suppress the gene expression patterns.

### Statistical analysis

The statistical analyses were all accomplished with R software (version 4.1.1). When comparing the gene expression levels, we applied Student’s t-test. The categorical variables were compared by the Pearson chi‐square test. The Kaplan–Meier curve was applied to compare the survival time and survival rates between subgroups. Multivariable Cox regression models were utilized to construct the predictive risk model in this study.

**FIGURE 1:**
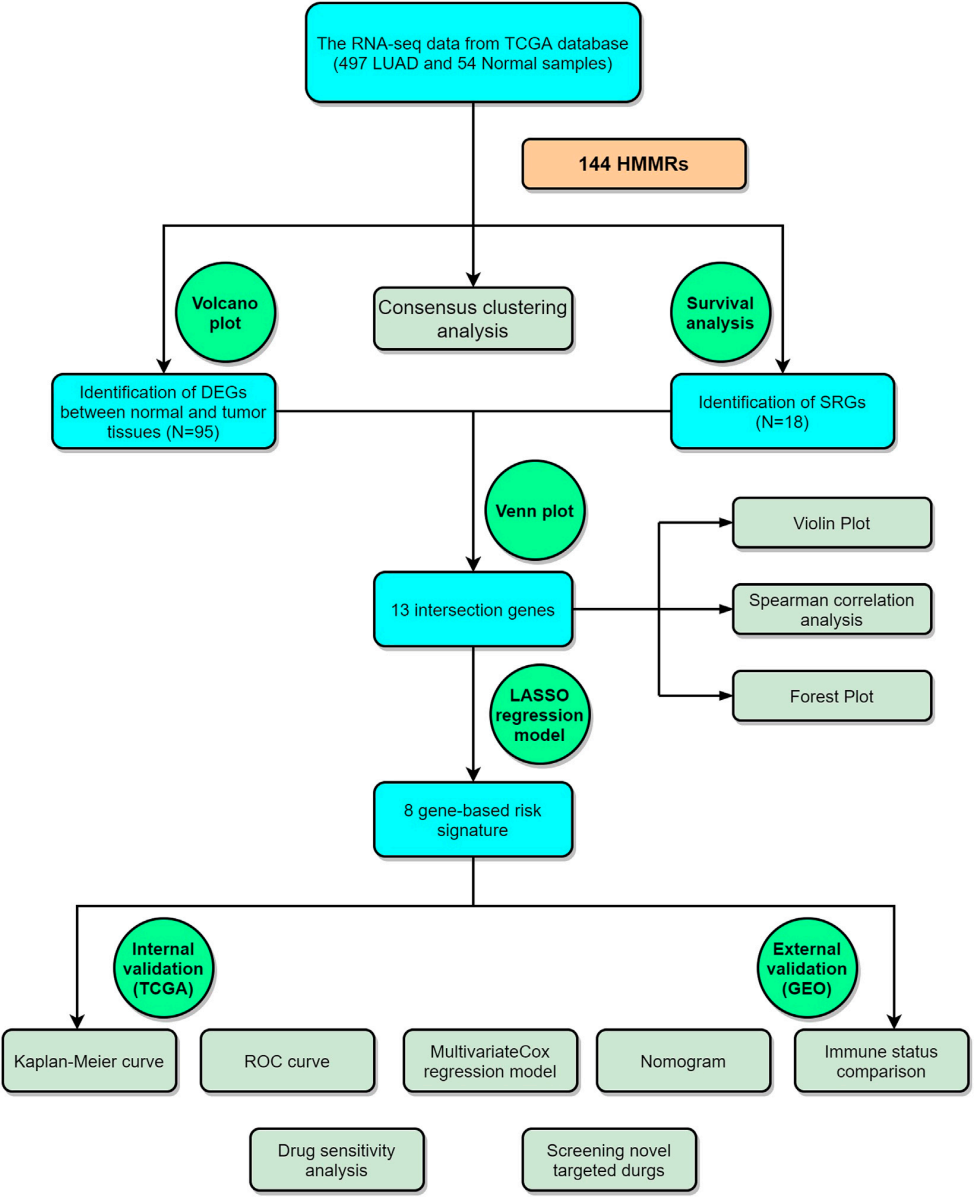
The graphical workflow maps of the study.

The analysis process of this study is shown in Figure 1.

## Results

### Consensus clustering of histone methylation modification regulators identified 2 subtypes of lung adenocarcinoma

**FIGURE 2:**
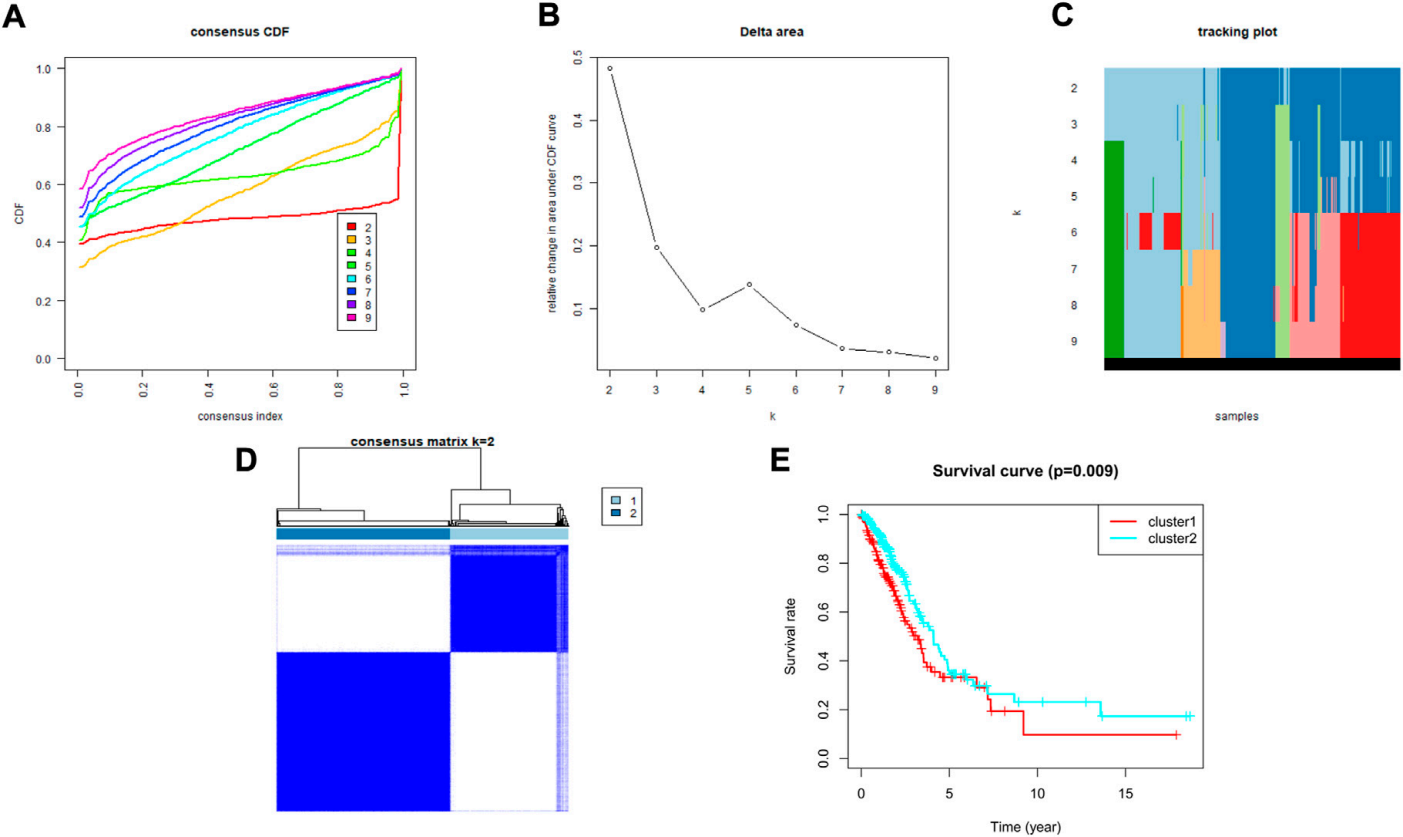
Tumor classification based on the HMMRs. **(A)** Consensus clustering cumulative distribution function (CDF) for k = 2 to 9. **(B)** The changes in the area under the CDF curve for k = 2 to 9. **(C)** The tracking plot for k = 2 to 9. **(D)** The optimal consensus clustering matrix when k = 2; **(E)** The Kaplan–Meier OS curves for the two clusters.

To explore the association between the expression profile of HMMRs and the prognosis of LUAD patients, we applied consensus clustering analysis, which could provide an unbiased way to group all LUAD patients (only 436 patients had complete clinical information) based on the expression profiles of the 144 HMMRs. When increasing the clustering stability (k) from 2 to 9, we found that k = 2 seemed to be the optimal selection according to the expression similarity of HMMRs (Figures 2A–D). We then applied the Kaplan–Meier curve to compare the OS rate between the 2 clusters (N_cluster1_ = 183, N_cluster2_ = 253), and a notably lower survival possibility was found in patients classified into cluster 1 (*p* = 0.009, Figure 2E). Combining the clinical characteristics and the expression profiles of HMMRs, we constructed heatmaps to explore the discrepancies between the 2 clusters. We could acquire from the heatmaps that patients in cluster 2 had longer survival times, higher survival rates, a larger number of women, earlier tumor stages, and fewer tumor metastases (Supplementary Figure S1). The details of the clinical features between the 2 clusters are included in Supplementary Table S2.

### Identification of the differentially expressed genes among 144 histone methylation modification regulators

**FIGURE 3:**
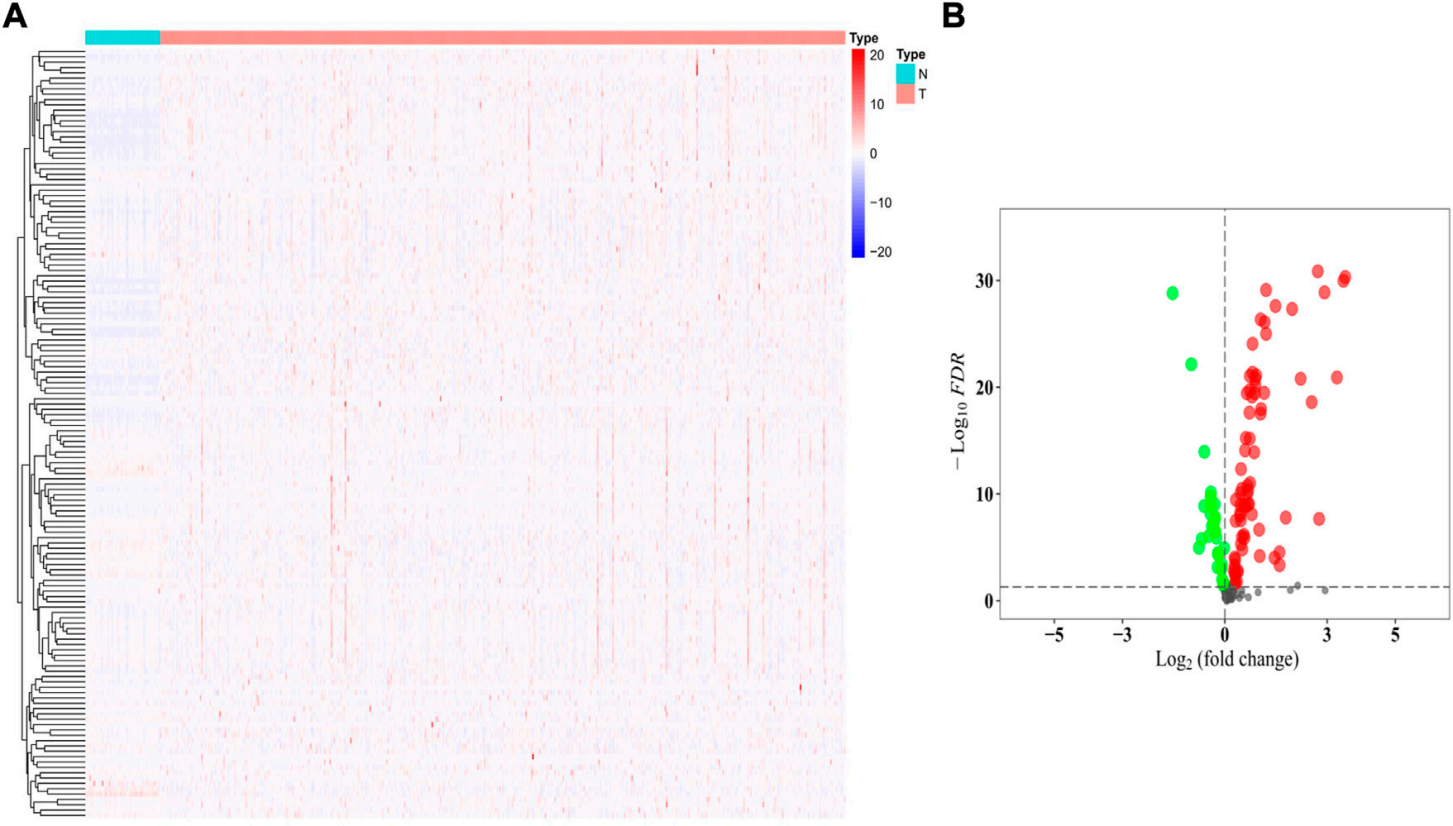
Identification of the DEGs between normal and tumor samples. **(A)** Heatmap of the 144 HMMRs between normal (N) and tumor tissues (T); **(B)** Volcano plot showing the DEGs (green dots: downregulated at least 2-fold in tumor tissues; red dots: upregulated at least 2-fold in tumor tissues).

The gene expression levels of the 144 HMMRs were compared between 54 normal lung samples and 497 LUAD specimens in the TCGA cohort, and the expression heatmaps are presented in Figure 3A. We found that most of the HMMRs were enriched in the tumor samples (Supplementary Table S3). Additionally, a volcano plot was also applied to show the expression differences (green plots: downregulated genes in tumors; red plots: upregulated genes in tumors, Figure 3B). The DEGs were screened out within the criteria of *FDR* < 0.01, and a total of 95 DEGs were identified.

### Exploration of the prognostic values of these histone methylation modification regulators

**FIGURE 4:**
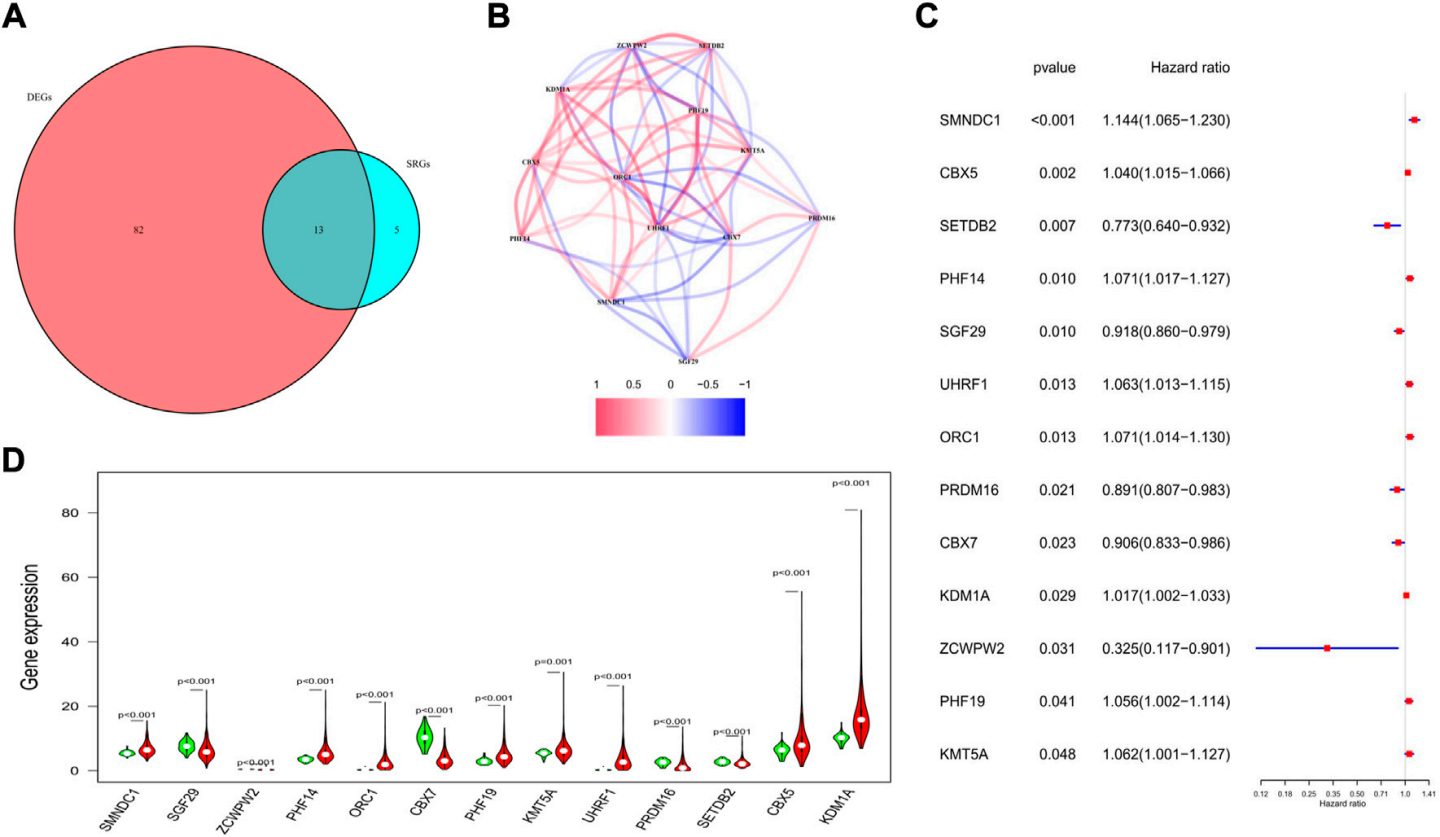
Characteristics of the 13 candidate genes. **(A)** Venn diagram showing the intersection genes between DEGs and SRGs. **(B)** Spearman correlation analysis for the 13 genes (red, positive correlations; blue, negative correlations). **(C)** Forest plot showing the prognostic value of the 13 genes. **(D)** Violin plot showing the expression levels of the 13 genes between normal (green) and tumor tissues (red).

Univariate regression analysis was applied to evaluate the prognostic values of the 144 HMMRs by combining the survival information and the expression level of each gene. Following the criteria of *p* < 0.05, 18 genes were screened out. Among them, 13 genes (*SMNDC1*, *CBX5*, *SETDB2*, *PHF14*, *SGF29*, *UHRF1*, *ORC1*, *PRDM16*, *CBX7*, *KDM1A*, *ZCWPW2*, *PHF19*, *KMT5A*) were also identified as DEGs between normal and tumor samples (Figure 4A), and the Kaplan-Meier curve for each gene was presented in Supplementary Figure S2. To investigate the association of the 13 key genes, we established a co-expression network according to the mRNA level of each gene in the TCGA cohort (red: positive correlation, blue: negative correlation, Figure 4B). We next utilized the forest plot to show the hazard ratio (HR) and the 95% confidence interval (95% CI) of the 13 hub genes (Figure 4C) while applying the violin plot to display the differential expression of each gene between normal and tumor tissues (Figure 4D). We confirmed that *SETDB2* (HR: 0.773, 95%CI: 0.640–0.932), *SGF29* (HR: 0.918, 95%CI: 0.860–0.979), *PRDM16* (HR: 0.891, 95%CI: 0.807–0.983), *CBX7* (HR: 0.906, 95%CI: 0.833–0.986), and *ZCWPW2* (HR: 0.325, 95%CI: 0.117–0.901) were protective genes, and they were all downregulated in the tumor samples.

### Development and validation of a prognostic gene signature

**FIGURE 5:**
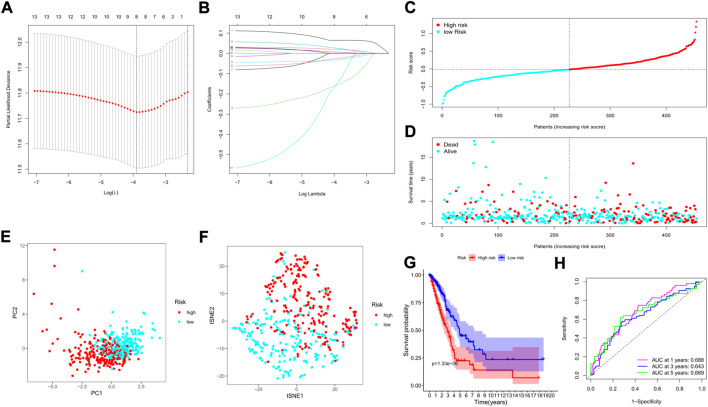
Construction of a risk model based on the TCGA cohort. **(A)** The cross-validation for tuning the parameter selection; **(B)** The LASSO regression for the 13 candidate genes; **(C)** The distribution of risk scores for the patients; **(D)** Survival status for each patient (low-risk: left of the dotted line; high-risk: right of the dotted line); **(E)** The PCA plot based on the risk groups; **(F)** The t-SNE analysis for the two risk groups; **(G)** The Kaplan–Meier curves to show the survival possibilities between low- and high-risk group; **(H)** The time-dependent ROC curves for 1-, 3-, and 5-years.

We applied the least absolute shrinkage and selection operator (LASSO) regression model based on the expression profiles of the 13 genes to construct our risk model. According to the optimum λ value (λ_min_ = 0.0223, left dotted line; λ_1se_ = 0.0989, right dotted line, Figure 5A), an 8-gene-based risk signature was finally established, and the coefficient of each gene was shown in Figure 5B. The risk score was calculated by the following formula (gene names mean the expression levels): risk score = 0.125*SMNDC1+0.107*CBX5+(−0.172)*SETDB2+0.073*PHF14+(0. 086)*SGF29+(−0.051)* *PRDM16*+(−0.030)* *ZCWPW2*+0.016* *PHF19*. Based on the formula, the risk score of each patient was determined and ranged from −0.990 to 1.342. Referring to the median risk score (−0.018), all patients in the training cohort were divided into low- and high-risk subgroups (Figure 5C). The distribution plot revealed that patients in the high-risk group suffered lower survival possibilities and shorter OS times (Figure 5D). Moreover, we applied PCA and t-SNE analysis to show the discrepancy in gene expression profiles, and the results showed clear separations between the 2 subgroups (Figures 5E,F). The Kaplan–Meier curve showed significantly lower survival rates and OS times in the high-risk subgroup (*p* < 0.001, Figure 5G). The time-dependent receiver operating characteristic (ROC) curve was applied to evaluate the sensitivity and specificity of the predictive model, and we observed that the area under the curve (AUC) was 0.688 for 1 year, 0.643 for 3 years, and 0.669 for 5 years (Figure 5H).

To clarify the relationships between the tumor subtypes and risk subgroups, we compared the risk scores and the numbers of high-risk patients between the 2 clusters. As it was shown in Supplementary Figure S3, the risk scores and the numbers of high-risk patient were much higher in cluster 1 than that in cluster 2 tumor type (*p* < 0.0001).

**FIGURE 6:**
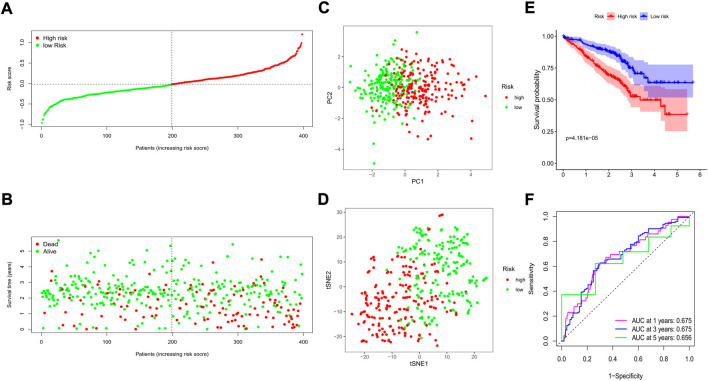
Validation of the prognostic value of the risk signature in an external GEO cohort. **(A)** The distribution of risk scores for each patient; **(B)** Survival status for each individual (low-risk: left of the dotted line; high-risk: right of the dotted line); **(C)** The PCA plot for the two risk groups; **(D)** The t-SNE analysis for the two risk groups; **(E)** The Kaplan–Meier curves to compare the OS time between the two risk groups; **(F)** The time-dependent ROC curves for 1-, 3-, and 5-years.

The GSE72094 GEO cohort containing 398 LUAD patients with complete clinical information was utilized as the external validation set. The risk score of each patient was calculated by the risk score formula, and according to the median risk score of the training cohort, 398 patients in the validation cohort were divided into either low-(N = 199) or high-risk (N = 199) groups (Figure 6A). We also discovered that the number of deaths was larger in the high-risk group (Figure 6B). Similarly, based on the gene expression profiles, patients in the 2 subgroups were well separated into two directions in the PCA and t-SNE plots (Figures 6C,D). The Kaplan–Meier analysis also indicated a significant difference in the survival time between the two risk subgroups (*p* < 0.001, Figure 6E). Moreover, the time-dependent ROC analysis showed that our risk model could be a favorable predictor in the validation cohort, and the AUC was 0.675 for 1 year, 0.675 for 3 years, and 0.656 for 5 years (Figure 6F).

### Independent prognostic value of the risk model

**FIGURE 7:**
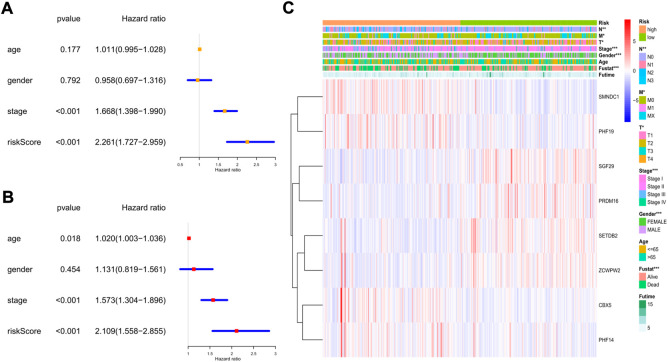
The independent prognostic value of the risk score in the training cohort. **(A)** The forest plot for univariate analysis; **(B)** The forest plot for multivariate analysis; **(C)** The heatmaps for the gene expression combined with clinical characteristics.

To explore whether the risk model could independently predict the prognosis of LUAD patients, we applied univariate and multivariate Cox regression models for validation. The clinical features (age, sex, and tumor stage) of each patient were extracted and applied in regression models. In the training cohort, we found that tumor stage and the risk score were risk factors associated with poor prognosis in the univariate regression model (Figure 7A). In the multivariate regression model, patient age (HR: 1.020, 95% CI: 1.003–1.036, *p* = 0.018), tumor stage (HR: 1.573, 95% CI: 1.304–1.896, *p* < 0.001), and risk score (HR: 2.109, 95% CI: 1.558–2.855, *p* < 0.001) were identified as independent risk factors (Figure 7B). Then, we constructed heatmaps to compare the differences in the clinical features and the expression of the 8 key genes between the low- and high-risk subgroups in the TCGA cohort, and we found that the mRNA levels of the key genes, tumor stage (including T, N, and M stages), sex, and survival status were quite different between the 2 clusters (Figure 7C).

**FIGURE 8:**
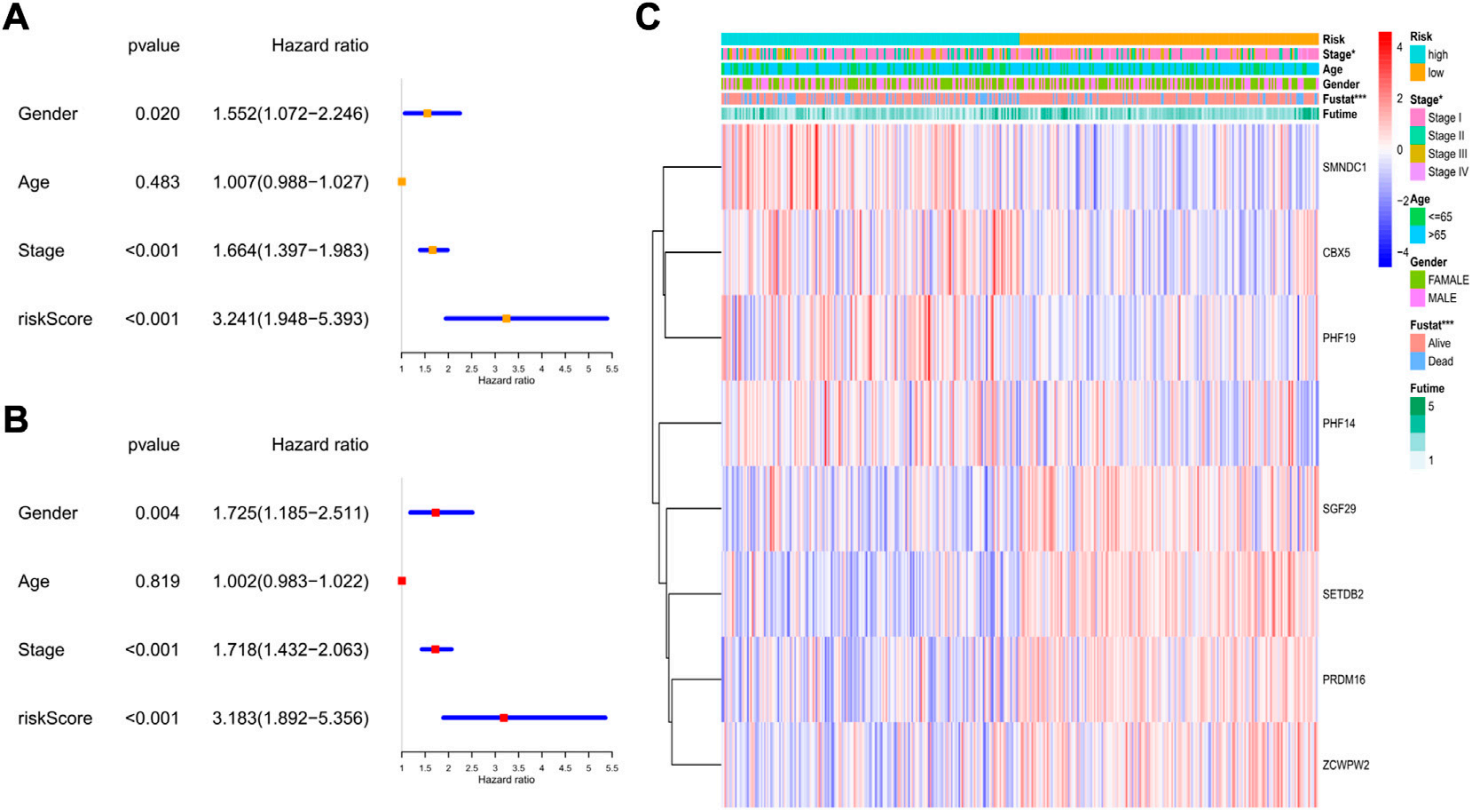
The independent prognostic value of the risk score in the validation set. **(A)** The forest plot for univariate analysis; **(B)** The forest plot for multivariate analysis; **(C)** The heatmaps for the gene expression combined with clinical characteristics.

In the GEO cohort, the univariate analysis indicated that sex, tumor stage, and risk score were risk factors (Figure 8A). In the multivariate model, we found that sex (HR: 1.725, 95% CI: 1.185–2.551, *p* = 0.004), tumor stage (HR: 1.781, 95% CI: 1.432–2.063, *p* < 0.001), and risk score (HR: 3.183, 95% CI: 1.892–5.356, *p* < 0.001) were also independent risk factors for prognosis (Figure 8B). The heatmap revealed that the tumor stage, survival status, and expression levels of the 8 genes were quite different between the two risk subgroups (Figure 8C).

**FIGURE 9:**
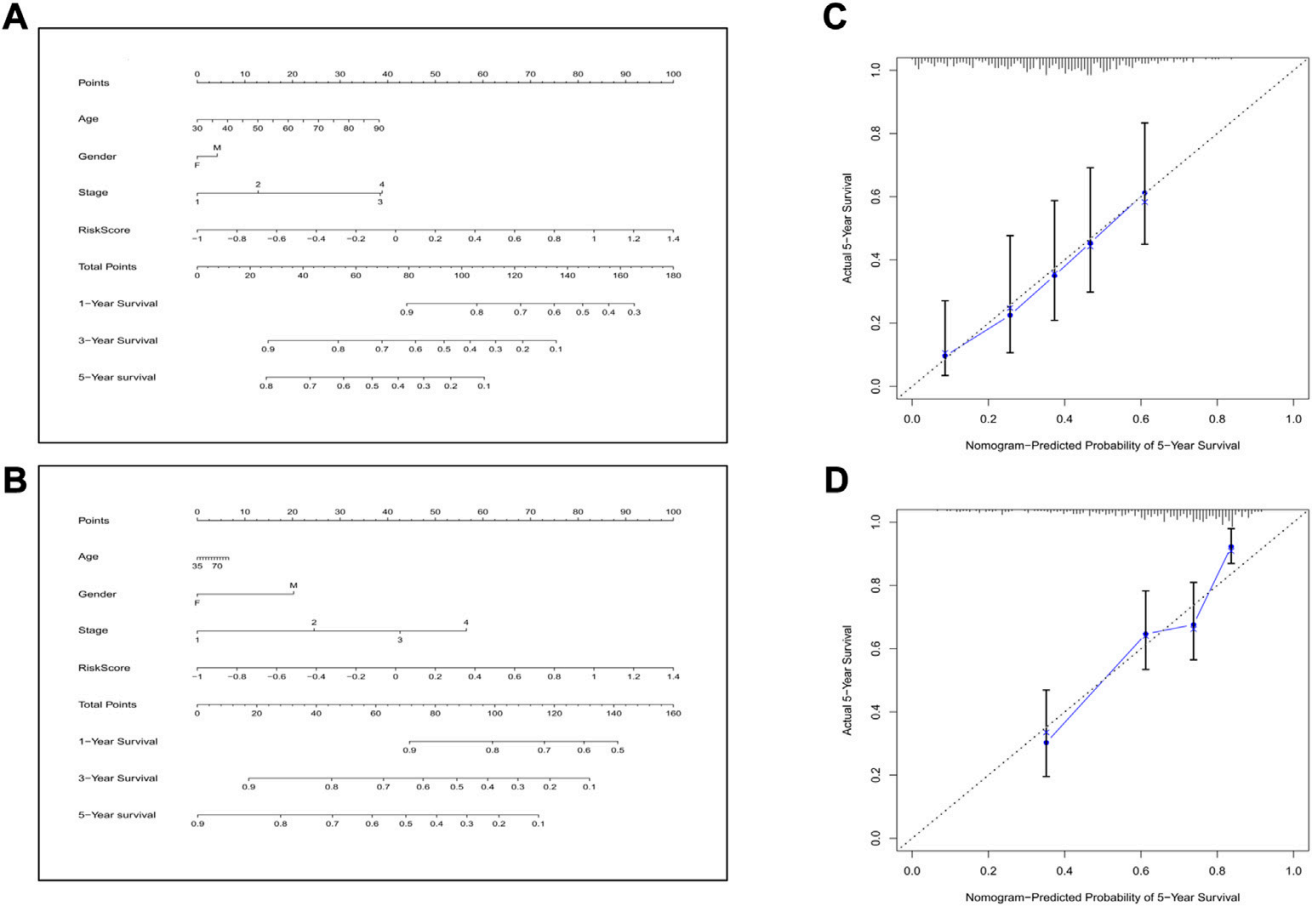
Construction of the nomogram and the calibration curve. **(A)** The nomogram for the training cohort. **(B)** The nomogram for the validation cohort. **(C)** The calibration curves for the model in the training set. **(D)** The calibration curves for the model in the validation set.

Next, we combined all the clinical features and the risk score to construct a predictive nomogram by applying the logistic regression model. In the TCGA cohort, the 1-, 3-, and 5-year survival rates could be well predicted (Figure 9A). We also established a nomogram in the GEO cohort, which is shown in Figure 9B. To validate the accuracies of the models, we employed the calibration curve, and the results indicated that our predictive models revealed high consistencies to ideal 5-year survival rates in both TCGA and GEO cohorts (Figures 9C,D).

### Comparison of immune cells and immune activities between the low- and high-risk groups

**FIGURE 10:**
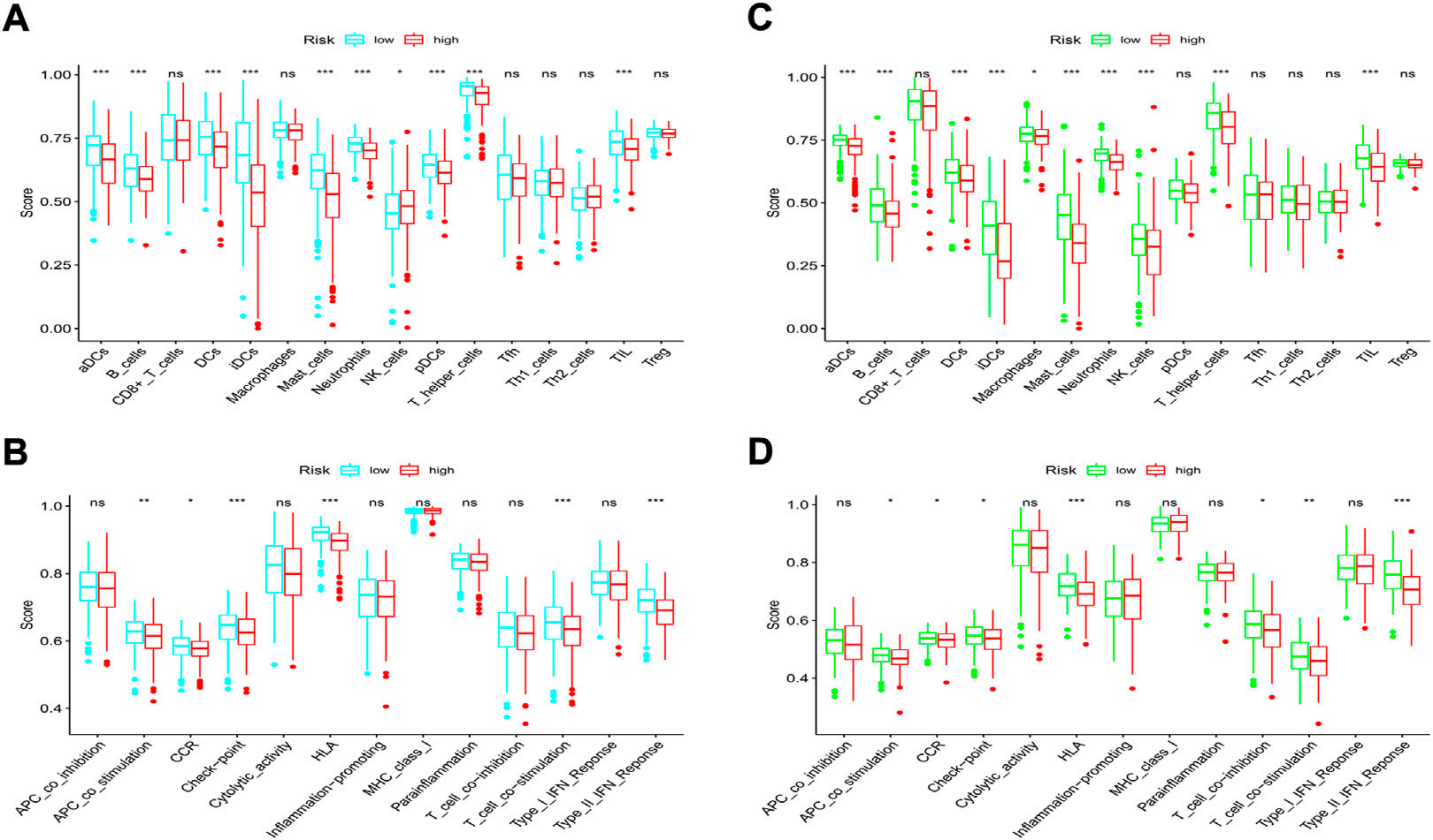
Comparison of immune cells and immune pathways. **(A,B)** Comparison of the enrichment scores of 16 immune cells and 13 immune-related pathways between the low- and high-risk groups in the training set. **(C,D)** Comparison of the enrichment scores of 16 immune cells and 13 immune-related pathways between the low- and high-risk groups in the validation set. (ns, not significant; *p < 0.05; **p < 0.01; ***p < 0.001).

According to the classical marker proteins of immune cells and the major genes participating in immune-related pathways, we identified 16 types of immune cells and 13 immune-related signaling pathways (Supplementary Table S1). We next established a scoring system by employing ssGSEA to evaluate the immune status among groups. In the TCGA cohort, most of the immune cells were at lower infiltration levels in the high-risk subgroup, especially B cells, all types of dendritic cells (DCs), mast cells, neutrophils, tumor-infiltrating lymphocytes (TILs), and T helper cells (all *p* < 0.001, Figure 10A). In addition, we discovered that immune-related signaling pathways were inactive in the high-risk group (Figure 10B). Similarly, in the GEO cohort, we also found that most of the immune cells were at lower levels and that the activity of the immune-related signaling pathways was impaired in the high-risk group (Figures 10C,D). Moreover, we noticed that the activities of the immune checkpoint pathways were decreased, indicating the confined antitumor effects of immune checkpoint inhibitors (ICIs) in the high-risk subgroup.

### GO and kyoto encyclopedia of genes and genomes analyses of differentially expressed genes between the low- and high-risk groups

**FIGURE 11:**
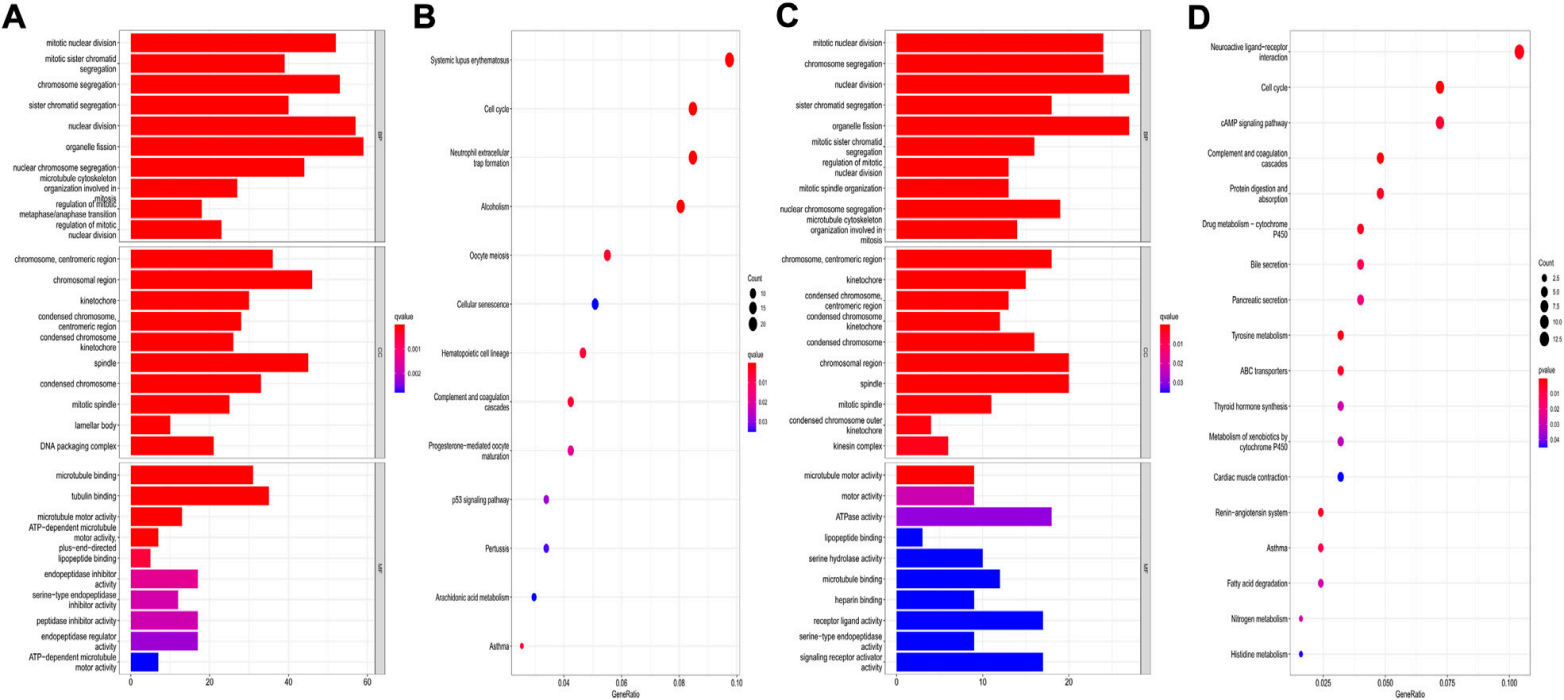
Functional analysis based on the DEGs between the two risk groups. **(A,B)** GO bar plot graph and KEGG bubble plot for the DEGs in the training cohort; **(C,D)** GO bar plot graph and KEGG bubble plot for the DEGs in the validation cohort. (q-value: the adjusted *p* value).

The “limma” R package was utilized to identify the DEGs between the low- and high-risk subgroups by following the criteria *FDR* < 0.05 and |log_2_FC | ≥ 1. Finally, 659 DEGs between the subgroups in the TCGA cohort were screened out, and among them, 343 genes were downregulated, while 316 were enriched in the high-risk group (Supplementary Table S4). Functional enrichment analyses were then set up based on these DEGs. The GO analysis showed that the DEGs mainly participated in the procedures of mitosis (Figure 11A). Moreover, the KEGG pathway analysis indicated that most of the DEGs were involved in systemic lupus erythematosus, the cell cycle, and neutrophil extracellular trap formation (Figure 11B). In the GEO cohort, we also found that the DEGs were related to the main steps of cell division by applying GO analysis (Figure 11C). The results of the KEGG analysis demonstrated that the DEGs were associated with neuroactive ligand–receptor interactions, the cell cycle, and the cAMP signaling pathway (Figure 11D). Accordingly, we can confirm that the DEGs between subgroups divided by our risk signature were closely related to the cell cycle.

### Drug sensitivity analysis and identification of promising therapeutic drugs for high-risk patients

**FIGURE 12:**
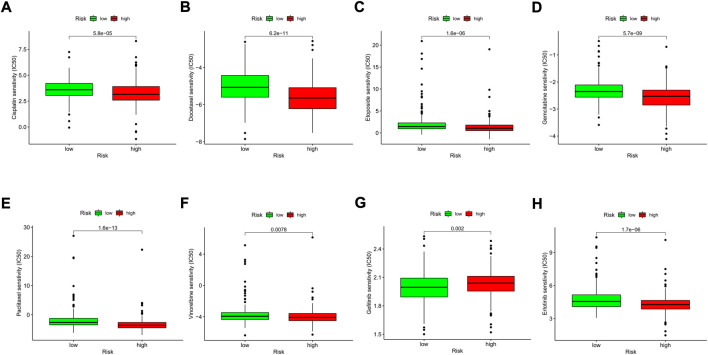
Comparison of the drug sensitivities between low- and high-risk LUAD patients. **(A–H)** Comparison of the IC50 of each drug between the two risk groups.

We explored the association between the risk model and the efficacy of the commonly used drugs in patients with LUAD. We listed 6 common chemotherapy drugs, including cisplatin, docetaxel, etoposide, gemcitabine, paclitaxel, and vinorelbine. Significant differences in the IC50 between the two risk groups were discovered (Figures 12A–F), suggesting that our risk model could be applied to predict the sensitivity of chemotherapy. Gefitinib and erlotinib are both first-generation EGFR-TKIs; however, there are still some controversies about the efficacy advantages of the two drugs. Our results indicated that the low-risk LUAD population was more sensitive to gefitinib, while erlotinib revealed more therapeutic advantages in high-risk LUAD patients (Figures 12G,H).

Then, we applied the cMap tool to screen the effective candidate drugs based on the DEGs between the low- and high-risk groups. According to the similarity scores (ranging from -100 to 100), we listed the top 20 antagonistic drugs in the TCGA cohort (Table 1). Meanwhile, the top 20 blockers based on the DEGs of the GEO cohort are presented in Table 2, and 12 of them were identified to be crossover drugs (purvalanol-a, JAK3-inhibitor-VI, aminopurvalanol-a, PHA-793887, camptothecin, AS-601245, topotecan, ZG-10, JNJ-7706621, dactinomycin, bisindolylmaleimide-ix, and A-443644). Consequently, these 12 drugs could be considered potential therapeutic agents for high-risk LUAD patients based on our risk model.

**TABLE 1:** The top 20 candidate drugs based on the DEGs in the TCGA cohort.

Score	ID	Name	Description	MOA
−99.82	BRD-K50836978	purvalanol-a	CDK inhibitor	CDK inhibitor, DYRK inhibitor
−99.4	BRD-K04546108	JAK3-inhibitor-VI	JAK inhibitor	JAK inhibitor
−99.15	BRD-K00615600	AG-14361	PARP inhibitor	PARP inhibitor
−98.91	BRD-K07762753	aminopurvalanol-a	Tyrosine kinase inhibitor	Tyrosine kinase inhibitor, CDK inhibitor
−98.77	BRD-K99749624	linifanib	PDGFR receptor inhibitor	PDGFR receptor inhibitor, VEGFR inhibitor
−98.63	BRD-K52522949	NCH-51	HDAC inhibitor	HDAC inhibitor
−98.52	BRD-A30437061	camptothecin	Topoisomerase inhibitor	Topoisomerase inhibitor
−98.45	BRD-K64800655	PHA-793887	CDK inhibitor	CDK inhibitor
−98.41	BRD-A60245366	AS-601245	JNK inhibitor	JNK inhibitor
−98.41	BRD-K06543683	bisindolylmaleimide-ix	CDK inhibitor	CDK inhibitor, PKC inhibitor
−98.38	BRD-K11636097	JNJ-7706621	CDK inhibitor	CDK inhibitor
−98.38	BRD-K69840642	ISOX	HDAC inhibitor	HDAC inhibitor
−98.38	BRD-A73909368	dactinomycin	RNA polymerase inhibitor	RNA polymerase inhibitor
−98.38	BRD-K43389675	daunorubicin	RNA synthesis inhibitor	RNA synthesis inhibitor, Topoisomerase inhibitor
−98.38	BRD-A02333338	cyclopamine	Smoothened receptor antagonist	Smoothened receptor antagonist
−98.34	BRD-U51951544	ZG-10	JNK inhibitor	JNK inhibitor
−98.34	BRD-K38615104	A-443644	AKT inhibitor	AKT inhibitor
−98.34	BRD-K12867552	THM-I-94	HDAC inhibitor	HDAC inhibitor
−98.31	BRD-K13566078	BMS-345541	IKK inhibitor	IKK inhibitor
−98.31	BRD-A59985574	topotecan	Topoisomerase inhibitor	Topoisomerase inhibitor

**TABLE 2:** The top 20 candidate drugs based on the DEGs in the GEO cohort.

Score	Id	Name	Description	MOA
−99.79	BRD-K50836978	purvalanol-a	CDK inhibitor	CDK inhibitor, DYRK inhibitor
−99.75	BRD-K04546108	JAK3-inhibitor-VI	JAK inhibitor	JAK inhibitor
−99.37	BRD-K07762753	aminopurvalanol-a	Tyrosine kinase inhibitor	Tyrosine kinase inhibitor, CDK inhibitor
−98.77	BRD-K56334280	amonafide	Topoisomerase inhibitor	Topoisomerase inhibitor
−98.45	BRD-K64800655	PHA-793887	CDK inhibitor	CDK inhibitor
−98.45	BRD-A30437061	camptothecin	Topoisomerase inhibitor	Topoisomerase inhibitor
−98.34	BRD-A60245366	AS-601245	JNK inhibitor	JNK inhibitor
−98.34	BRD-A59985574	topotecan	Topoisomerase inhibitor	Topoisomerase inhibitor
−98.31	BRD-U51951544	ZG-10	JNK inhibitor	JNK inhibitor
−98.31	BRD-K92093830	doxorubicin	Topoisomerase inhibitor	Topoisomerase inhibitor
−98.24	BRD-K11636097	JNJ-7706621	CDK inhibitor	CDK inhibitor
−98.21	BRD-K87909389	alvocidib	CDK inhibitor	CDK inhibitor
−98.2	BRD-K99545815	PF-562271	Focal adhesion kinase inhibitor	Focal adhesion kinase inhibitor
−98.17	BRD-A11702965	chromomycin-a3	DNA binding agent	DNA binding agent
−98.17	BRD-K19220233	JNK-9L9 L	JNK inhibitor	JNK inhibitor
−98.17	BRD-K13390322	AT-7519	CDK inhibitor	CDK inhibitor, Cell cycle inhibitor
−98.17	BRD-A73909368	dactinomycin	RNA polymerase inhibitor	RNA polymerase inhibitor
−98.13	BRD-K06543683	bisindolylmaleimide-ix	CDK inhibitor	CDK inhibitor, PKC inhibitor
−98.1	BRD-K38615104	A-443644	AKT inhibitor	AKT inhibitor
−98.03	BRD-K79090631	CGP-60474	CDK inhibitor	CDK inhibitor

## Discussion

In this study, we first explored the associations between HMMRs and LUAD. According to the gene expression patterns of the 144 HMMRs, all LUAD individuals could be separated into 2 tumor subtypes, and a significant difference in the survival rate was observed between the 2 subtypes, indicating that these regulators play important roles in the development of LUAD. Ninety-five of the HMMRs were then identified as DEGs between LUAD and normal lung samples, and 13 of them were further investigated as SRGs. By employing the LASSO Cox regression model, we constructed an 8-gene risk signature in the TCGA cohort, which was verified to have favorable prognostic value in both the training and validation cohorts. LUAD patients in each cohort were classified into 2 risk groups based on our risk model, and we found that in the high-risk group, immune cells were less abundant, while immune activities were decreased compared with those in the low-risk group. Functional analysis indicated that the DEGs between the low- and high-risk groups were mainly related to the cell cycle. We next conducted a drug sensitivity analysis, and the results indicated that our risk model could be applied to predict the sensitivity of commonly used chemotherapeutic drugs. With the help of the cMap tool, we discovered 12 new drugs that could be potential therapeutic agents for high-risk LUAD patients.

According to the specific functions in histone methylation, the 8 genes in our risk signature could be classified into 2 types: *PRDM16* and *SETDB2* belong to the HMTs, while *SMNDC1*, *PHF19*, *SGF29*, *ZCWPW2*, *CBX5*, and *PHF14* appertain to the HMRPs. Among them, *PRDM16*, *SETDB2*, *SGF29*, and *ZCWPW2* were protective genes and were all downregulated in the tumor samples, while the others were enriched in the cancer tissues and were associated with poor prognosis. PR domain-containing 16 (*PRDM16*) is a member of the PRDM family, which contains a conserved PR structure and multiple zinc finger structures at its N-terminal end and has been proven to catalyze the mono-methylation of H3K9 (Biferali et al., 2021). *PRDM16* was initially found to enhance the function of brown adipocytes, promote their differentiation, induce the conversion of precursor adipocytes into brown adipocytes and promote the differentiation and maturation process of brown adipocytes (Chi and Cohen, 2016), while its role in tumors has been less studied. Our results demonstrated that *PRDM16* functioned as a tumor suppressor gene, and this may be explained in Fei et al.‘s study, which showed that *PRDM16* could regulate histone methylation in the promoter region of *MUC4* to reduce its expression and inhibit the EMT process mediated by *MUC4* (Fei et al., 2019). *SETDB2* (SET domain bifurcated histone lysine methyltransferase 2) contains a bifurcated SET region, an anterior SET region, and a methylated CpG-binding region that can trimethylate H3K9. A recent review reported that low expression of *SETDB2* was associated with shorter disease-free survival time in renal cell tumors, while in gastric cancer, *SETDB2* overexpression predicted poor prognoses and was associated with tumor progression (Torrano et al., 2019). In our study, *SETDB2* was found to be downregulated in tumor samples, and its higher expression level predicted a better prognosis, indicating that it is a tumor suppressor gene. However, due to the rarity of studies on *SETDB2*, its specific mechanisms in LUAD still need further exploration. Survival motor neuron domain containing 1 (*SMNDC1*) was reported to be the “reader” of the asymmetrically deposited dimethylation at histone H3 arginine 17 (H3R17me2a) (Yang et al., 2010). It has been reported that silencing *SMNDC1* can significantly inhibit the proliferation of ovarian cancer cells, whereas the mechanism in ovarian cancer and the roles of *SMNDC1* in other tumors have not been elucidated (Giri et al., 2014). *SMNDC1* was identified as an ideal marker predicting poor prognosis in LUAD in our study, as it was upregulated in tumor samples and in high-risk LUAD populations. *PHF19* (PHD finger protein 19) recognizes the trimethylation of lysine 36 and lysine 27 on histone H3 (H3K36me3 & H3K27me3) through its own Tudor domain and is involved in chromosome activation (Dong et al., 2020). *PHF19* was discovered as a tumor-promoting gene in various cancers, including LUAD (Zhu et al., 2021), but the in-depth molecular mechanism has yet to be studied. SAGA-associated factor 29 (*SGF29*) has two tandem Tudor structural domains at the carboxyl terminus, which can recognize H3K4me2/3 and then lead to H3 acetylation and deubiquitination (Lu and Wang, 2013). Murakami et al. found that *SGF29* could enhance c-Myc-mediated malignant transformation (Murakami et al., 2014); nevertheless, our data revealed that it is downregulated in tumor samples, while its enrichment predicts better clinical outcomes, indicating that it is a tumor suppressor gene. Given the contradictory roles of *SGF29* in cancers, it is worth initiating further studies. *ZCWPW2* (zinc finger CW-type PWWP domain protein 2), which is the reader of H3K4me3 (Danuta et al., 2020), was identified as an antioncogene in our study. Until now, the functions of *ZCWPW2* in tumors have not been fully studied. Fan et al. revealed that a lower H3K4me3 level was associated with a poor prognosis for LUAD patients (Fan et al., 2022), and we hypothesized that *ZCWPW2* could maintain a higher level of H3K4me3 to play a tumor-suppressive role. Chromobox protein homolog 5 (*CBX5*), which has been proven to be associated with silenced, heterochromatic regions of the genome and belongs to the reader of H3K9me3. Yu et al. also identified *CBX5* as a tumor-promoting gene, and knockdown of *CBX5* decreased the aggressiveness of tumor stem-like cells (Yu et al., 2012). It has been reported that *CBX5* and H3K9me3 are enriched in the *FAS* and *PUMA* promoters in glioma, indicating that *CBX5* could suppress apoptotic activators by sustaining the methylation level of H3K9me3 (Lai et al., 2017). Further studies are necessary to clarify whether the mechanisms of the tumorigenic effect of *CBX5* in LUAD are similar to those in glioma. *PHF14* (PHD finger protein 14) is a conserved multi-PHD finger protein that can recognize unmodified lysine or arginine residues, dimethylated or trimethylated lysine residues, and acetylated lysine residues. However, the histone methylation-specific recognition sites of *PHF14* have remained indeterminate in the past few years. Recently, a study conducted by Zheng et al. revealed that *PHF14* was able to recognize H3K4me3 and H3R8me2a to exert its repressive functions (Zheng et al., 2021). In colorectal cancer, downregulation of *PHF14* could reduce carcinogenesis, and our study also indicated that *PHF14* was enriched in tumor samples and was associated with poor prognosis, while the molecular mechanisms were not clear. Further in-depth studies on *PHF14* should be considered.

Different sites and statuses of histone methylation can evolve many patterns of methylation modification, increasing the complexity and diversity of gene expression regulated by histone methylation. Generally, HMTs and HDMs carefully maintain histone methylation levels, whereas histone methylation is a relatively minor modification compared to other modifications and does not affect the binding of histones to DNA to any great extent. Therefore, most of the biological effects of histone methylation are thought to be mediated by highly specific HMRPs (Musselman et al., 2012). In addition, misinterpretation of histone methylation marks (abnormal HMRP activity) has been proven to be associated with many human diseases, including developmental abnormalities as well as cancer. Based on that, it is not hard to understand why most of the prognosis-related genes were the HMRPs in our study. HMRPs mediate a variety of roles, such as recruiting enzyme complexes, and can act as transcription factors or as other effector proteins. As the biological effects of HMRPs and their role in different tumors have been gradually revealed, HMRPs are becoming very attractive drug targets; however, structure-based drug design for targeting HMRPs is still in its infancy. Most of the current studies mainly focused on the predictive roles of HMTs and HDMs, neglecting the importance of HMRPs. Our work comprehensively explored the prognostic values of these HMMRs, providing insights and novel molecular targets for epigenetic therapy in LUAD.

In addition to providing prognostic values for LUAD patients, our risk model can also predict the efficacy of commonly used chemotherapy drugs. Previous research has indicated that the levels of histone methylation modification are related to the drug sensitivity of tumors. Wang et al. found that the transcription factor C/EBPβ contributes to increased H3K79 methylation modifications through recruitment of *DOT1L*, thereby reducing the efficacy of ovarian cancer chemotherapy (Wang et al., 2019). In NSCLC, researchers discovered that the levels of H3K27me3 and H3K4me3 could also be applied to determine the sensitivity of chemotherapies (Ávila-Moreno et al., 2014). In this work, the high-risk LUAD population was more sensitive to chemotherapies, demonstrating its higher degree of malignancy, and it may be related to the abnormal expression of these oncogenes regulated by the methylation of histones. Most of the clinical trials (Urata et al., 2016; Yang et al., 2017; He et al., 2021) showed that there were no differences in the therapeutic effects between gefitinib and erlotinib for EGFR-mutated NSCLC patients, complicating the choice of optimum drugs. Our results suggest that gefitinib is suitable for low-risk patients, while for high-risk LUAD patients, it is better to take erlotinib.

When comparing the immune status of the 2 subgroups, universally decreased activities were observed in the high-risk group. It is worth noting that the immune checkpoint pathways were inactive in the high-risk group, indicating that ICIs may have lower therapeutic efficiency. Additionally, the decreased amount of DCs, CD4^+^ T cells (T-helper cells), and TILs, combined with the impaired functions of antigen presentation, also revealed that the response to immunotherapy would not be good in the high-risk group. Previous studies have shown that chemotherapy-induced cell death can enhance the immunogenicity of tumors (Heinhuis et al., 2019), activating APC functions, while the inflammatory response generated by cell death can upregulate PD-L1 expression (Zhang et al., 2016), and both can synergistically enhance the efficacy of immunotherapy. Based on the current evidence and our findings, a high-risk population should be given priority to chemotherapy, and then followed by immunotherapy, while erlotinib should be chosen for those with EGFR-mutated high-risk LUAD patients.

The DEGs between the low- and high-risk groups were mainly enriched in the cell cycle pathways, and based on that, we screened several compounds that showed high curative possibilities for high-risk LUAD patients. However, explorations of most of these candidate drugs have not even been initiated in lung cancers, and our study may be of guiding significance in developing new targeted drugs against high-risk LUAD.

## Conclusion

Our study showed that histone methylation is closely connected to LUAD because most of the regulators are expressed differently between normal and tumor tissues. According to the histone methylation modification modes, LUAD can be divided into 2 tumor subtypes, which have distinctly different clinical features. We constructed a novel risk model based on the 8 HMMRs, and this model was proven to be an independent prognostic factor in both the training and validation cohorts, providing a new strategy for treating LUAD. In addition, our risk model can predict the efficacies of chemotherapy, EGFR-targeted therapy and immunotherapy and provides a theoretical basis for the development of new targeted drugs for LUAD.

## Data Availability

The datasets presented in this study can be found in online repositories. The names of the repository/repositories and accession number(s) can be found in the article/Supplementary Material.

## Supplementary material

SUPPLEMENTARY FIGURE S1
The heatmap and the clinicopathologic characteristics of the two clusters classified by these HMMRs.

SUPPLEMENTARY FIGURE S2
The Kaplan-Meier curves for the 13 intersection genes.

SUPPLEMENTARY FIGURE S3
Connections between tumor subtypes and risk subgroups. **(A)**Comparison of the risk scores between the 2 clusters. **(B)** Comparison of the risk populations between the 2 clusters.
