# Supplementary figures and images for: Comprehensive analysis of histone methylation modification regulators for predicting prognosis and drug sensitivity in lung adenocarcinoma
Source: Front Cell Dev Biol. 2022 Oct 3;10:991980. doi: 10.3389/fcell.2022.991980 (PMC9574078; doi:10.3389/fcell.2022.991980)

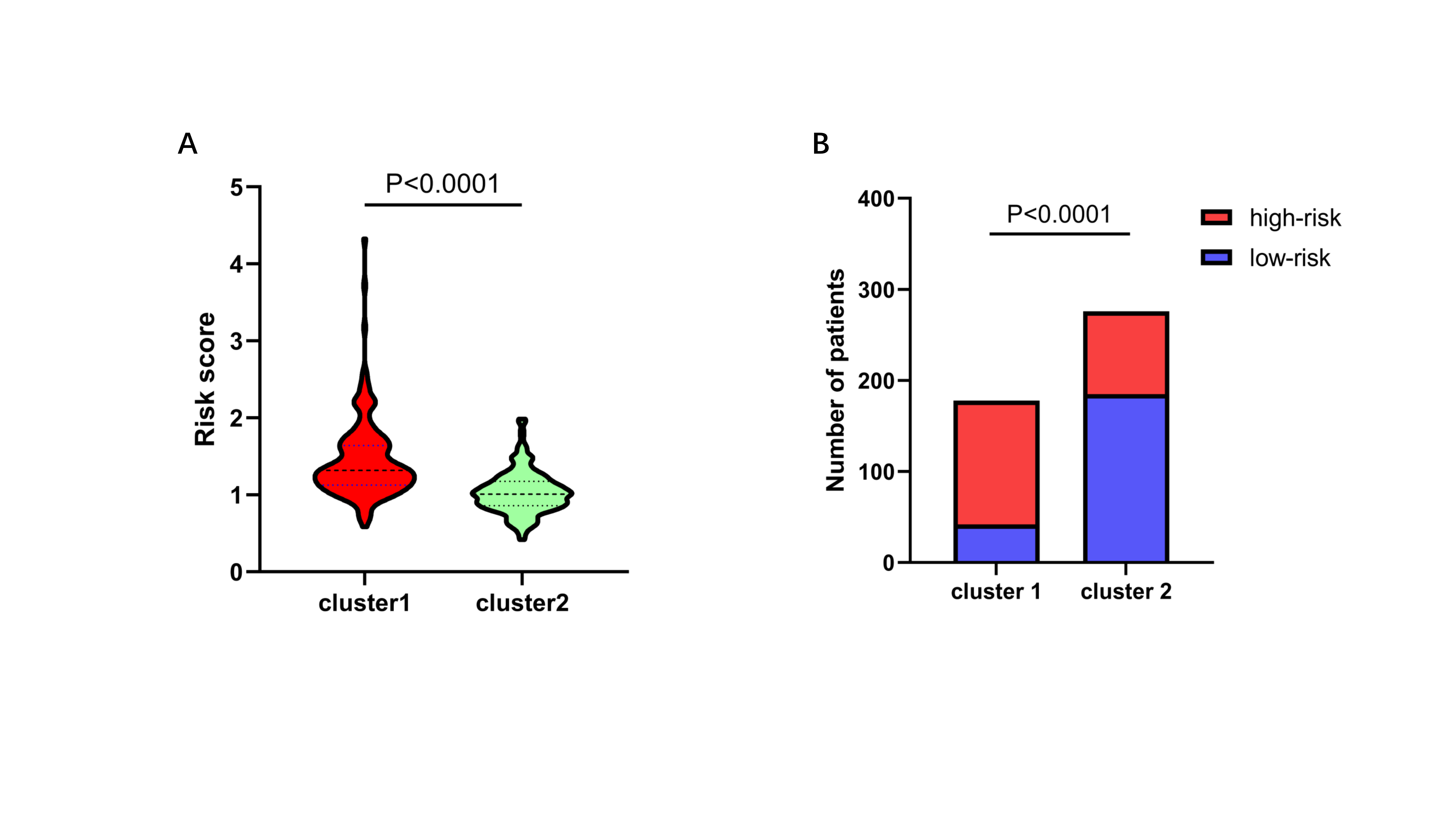

Supplement: Supplementary file 1 [file Image3.TIF]

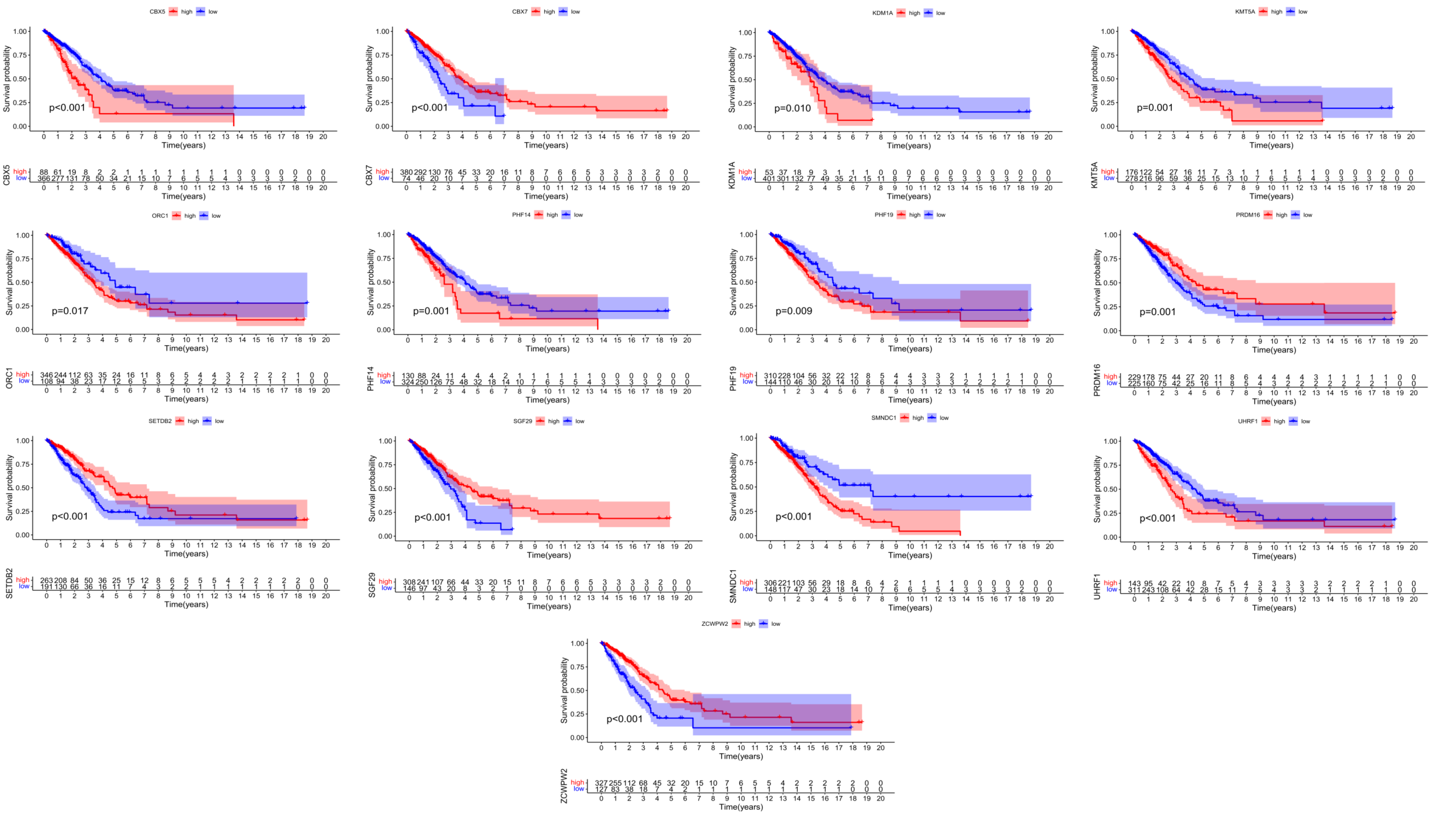

Supplement: Supplementary file 3 [file Image2.TIF]

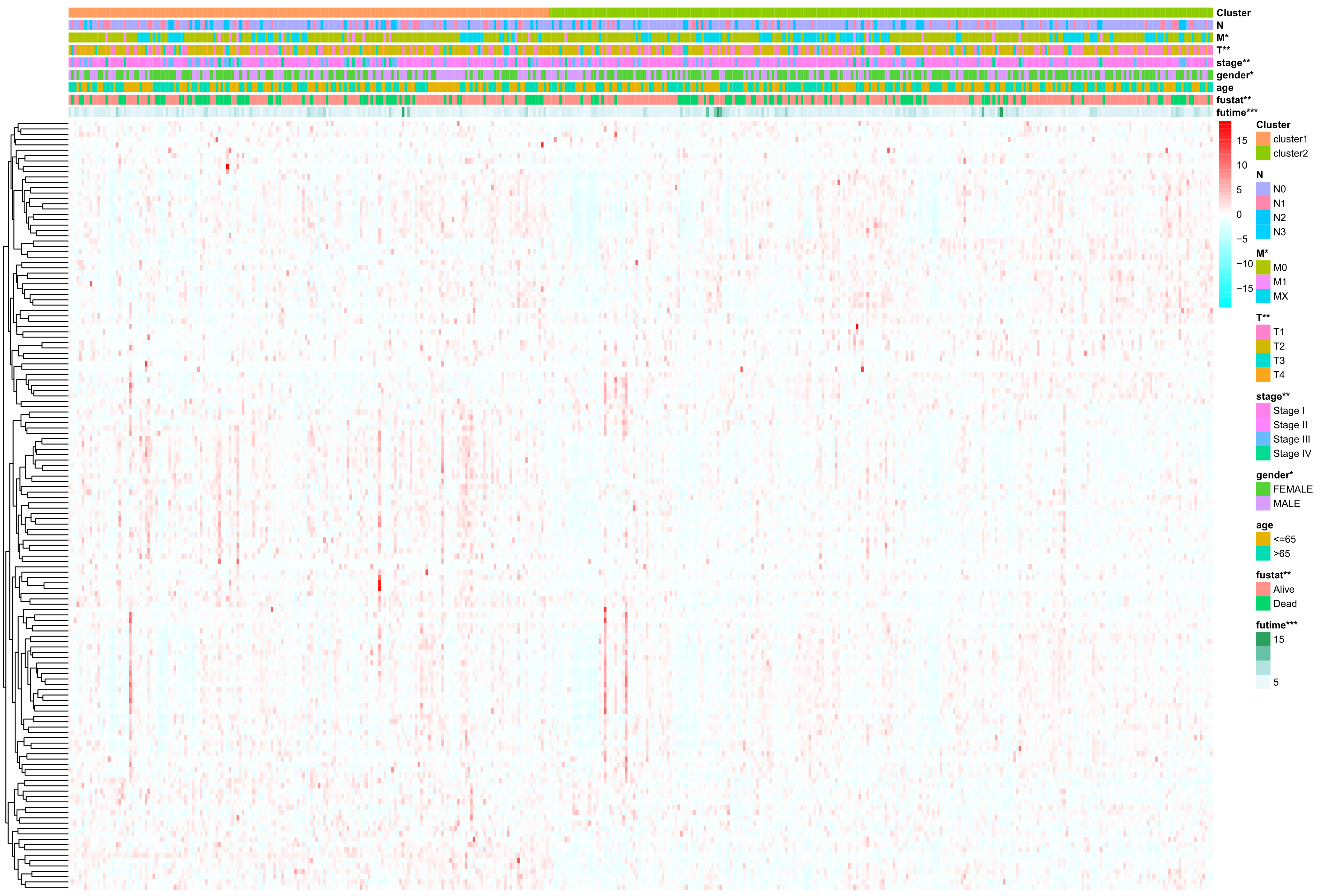

Supplement: Supplementary file 4 [file Image1.TIF]
